# Soil Fungal Community Composition and Diversity of Culturable Endophytic Fungi from Plant Roots in the Reclaimed Area of the Eastern Coast of China

**DOI:** 10.3390/jof8020124

**Published:** 2022-01-27

**Authors:** Fei Zhong, Xinlei Fan, Wenhui Ji, Zhixing Hai, Naican Hu, Xintong Li, Guoyuan Liu, Chunmei Yu, Yanhong Chen, Bolin Lian, Hui Wei, Jian Zhang

**Affiliations:** 1 School of Life Science, Nantong University, Nantong 226019, China; 2108310001@stmail.ntu.edu.cn (W.J.); 1909110141@stmail.ntu.edu.cn (Z.H.); 1909110100@stmail.ntu.edu.cn (N.H.); 2009110009@stmail.ntu.edu.cn (X.L.); guoyuanliu@ntu.edu.cn (G.L.); ychmei@ntu.edu.cn (C.Y.); chenyh@ntu.edu.cn (Y.C.); lianziadd9@163.com (B.L.); weihui2021@ntu.edu.cn (H.W.); 2 Key Lab of Landscape Plant Genetics and Breeding, Nantong 226019, China; 3 The Key Laboratory for Silviculture and Conservation of the Ministry of Education, Beijing Forestry University, Beijing 100083, China; xinleifan@bjfu.edu.cn

**Keywords:** the reclaimed land, fungal community composition, ectomycorrhizal fungi, arbuscular mycorrhizal fungi, endophytic fungi

## Abstract

As an important resource for screening microbial strains capable of conferring stress tolerance in plants, the fungal community associated with the plants grown in stressful environments has received great attention. In this study, high-throughput sequencing was employed to study the rhizosphere fungal community in the reclaimed area (i.e., sites F, H, and T) of the eastern coast of China. Moreover, endophytic fungi from the root of six plant species colonizing the investigated sites were isolated and identified. The differences in soil physicochemical parameters, fungal diversity, and community structure were detected among the sampling sites and between the seasons. Ectomycorrhizal (ECM) fungi (e.g., genera *Tuber* and *Geopora*) were dominant at site F, which was characterized by high soil total carbon (SC) and total nitrogen (SN) contents and low soil electrical conductivity (EC) value. Arbuscular mycorrhizal (AM) fungi, including genera *Glomus*, *Rhizophagus*, and *Entrophospora* were dominant at sites H (winter), H (summer), and T (summer), respectively. The positive relationship between the EC value and the abundance of genus *Glomus* indicated the ability of this AM fungus to protect plants against the salt stress. Endophytic fungi at sites F (*Aspergillus* and *Tetracladium*), H (*Nigrospora*), and T (*Nigrospora*, *Coniochaeta* and *Zopfiella*) were recognized as the biomarkers or keystone taxa, among which only genus *Aspergillus* was isolated from the plant roots. The aforementioned AM fungi and endophytic fungi could contribute to the promotion of plant growth in the newly reclaimed land.

## 1. Introduction

The coastal area (Yellow Sea) in Jiangsu province of China has a great length of coastline (1039.7 km) and a large mudflat area (6520.6 km^2^) [1]. Large-scale coastal reclamation activities have been performed in China since 1950 to mitigate the conflict between the growing population and shrinking usable land [2]. Reclamation of coastal areas for agriculture, aquaculture, and forestry was considered as the preferred strategy to increase the food supply and improve the eco-environment [3]. So far, lots of reclamation activities have been done. However, the process of plants development in the reclaimed area is lagging behind due to some abiotic factors, among which saline stress is the most detrimental to plant growth [4]. Therefore, it is difficult to promote the exploitation and utilization of the reclaimed area, and more attention should be paid to improve saline stress tolerance of plants.

In saline habitats, only the halophytes that constitute about 1% of the world’s flora can thrive, contributing important eco-functions in the desert and coastal areas [5]. Halophytes are capable of coping with multiple environmental stresses, such as high salinity, tidal flooding, and nutrient deprivation [6,7]. Along with the reclamation processes, the vegetation will gradually shift from halophytes to non-halophytes (salt tolerant grasses, shrubs, and trees) in the old reclaimed regions. In a preliminary investigation in the reclaimed area, *Salix* was observed as the only tree species growing in all the investigated sites. As a pioneer tree species, willow could naturally grow in harsh soil conditions, which was partly ascribed to the development of dual mycorrhizal symbiosis (i.e., forming both arbuscular mycorrhizas and ectomycorrhizas within the same root system) [8]. The ability of some tree species (e.g., genera *Alnus*, *Eucalyptus*, *Populus* and *Salix*) to form dual mycorrhizal symbiosis is recognized as a key factor to improve their adaptions to unfavorable habitat conditions (e.g., the fluctuations of soil temperature, water content, salinity, and nutrients) [9,10]. It is necessary to explore the fungal community associated with plants (e.g., *Salix*) that survived or thrived in the reclaimed area.

Great attention has been put on the soil fungal communities, which maintains the ecosystem balance by contributing to the soil organic matter decomposition and mineralization [11]. It is noteworthy that some types of fungi in soil could penetrate into the plant tissue through the roots and wounds or by horizontal transmission through spores, forming extensive symbiotic relations with the host plant [12]. Being the two major groups of root fungal symbionts, mycorrhizal and endophytic fungi are generally believed to be critically important to improve host fitness, including productivity and abiotic tolerance, especially under stressful conditions [13,14]. There is relatively limited information concerning the role of the ectomycorrhizal (ECM) symbiosis in enhancing salt tolerance of plants. In contrast, considerable evidence indicates that arbuscular mycorrhizal (AM) fungi colonization in plant roots augments water and nutrient uptake capacities and enhances plant resistance to salinity stresses [15]. Unfortunately, AM fungi can hardly be grown in pure culture to obtain a large amount of inoculum, which restrained its large-scale application into integrated management of plants development in harsh environments [16,17]. Instead, endophytic fungi that can easily meet the requirements of mass production are gradually recognized as a highly promising mutualistic partners of plants. Endophytic fungi benefit their hosts by promoting resistance against high salinity stress via acting as elicitors in the process of resistance induction [18]. Nevertheless, endophytic fungi are known to shift their functional role between pathogenicity and mutualism depending on fungal genotype, host, and abiotic conditions [19]. The available information concerning the diversity of endophytic fungi and their potential function is limited, which deserves extensive investigation.

The research on plant-associated fungi in the coastal area has attracted global attention [20,21,22,23]. Most of the studies on fungi in the coastal area of China focused on mangroves ecosystems along the south China coast [24] and the salt marsh ecosystem in the Yellow River Delta [25]. Along the east China coast, limited information was obtained concerning either the root endophytic fungi or the soil fungi community in the reclaimed area. Moreover, the relationship between the soil fungal communities and the endophytic fungi is so complicated [26] that it deserves further investigation. The aims of this study include: (i) investigate the fungal community composition in the reclaimed regions and discover the critical factor mediating fungal genera succession; (ii) explore the potential relationship between the fungi in rhizosphere soil and the root endophytic fungi; (iii) examine the functional microbial taxa of the soil fungal community in the reclaimed regions. Identifying fungal community composition in the rhizosphere of the plants survived in the reclaimed area will be helpful in understanding the development of the host plants and their co-evolution with symbionts. Furthermore, collecting the isolated root endophytic fungi and constructing a fungal resource bank can pave the way for the ecosystem reconstruction of the reclaimed area along the east China coast.

## 2. Materials and Methods

### 2.1. Study Sites and Sampling

In two seasons (summer and winter), sites F (32.603° N, 120.880° E), H (32.609° N, 120.933° E), and T (32.155° N, 121.475° E) located in Nantong, Jiangsu province of China were chosen for the investigation of fungal community. The average annual precipitation and temperature were 1399.1 mm and 16.6 °C, respectively. Site T (close to the sea embankment) located in a region that was reclaimed recently, while site F (with a longer age of land reclamation) situated in a coastal shelter forest along the highway. In the middle of sites T and F, site H that was close to a farm was selected. At site H, the plants including *Salix matsudana*, *Tamarix chinensis*, *Suaeda glauca*, *Celtis sinensis*, and *Pyrus betulifolia* were distributed. At site T, *Salix matsudana* and *Tamarix chinensis* were observed in summer and winter, respectively. At site F, *Salix matsudana* was the dominant species in the shelter forest. The willows survived at sites F, H, and T for around 5, 3, and 2 years, respectively. Three 10 m × 10 m plots near the willows at each site were randomly selected for soil sampling. In each plot, five subsamples were collected and then mixed to make a composite soil sample. The rhizosphere soil was sampled using a shovel, and the plant roots below the ground was collected. The plant material and soil samples were transported to the laboratory and stored in a refrigerator for further analysis.

### 2.2. Physicochemical Analysis of the Soil Samples

The physicochemical parameters of the soil samples, including total carbon (SC), total nitrogen (SN), pH, and electrical conductivity (EC) were measured. Soil SC and SN were identified by combustion using a Vario EL Cube elemental analyzer (Elementar Ltd., Frankfurt, Germany). Soil pH and EC values was measured in 1:5 (*v/v*) soil/water extracts by a portable meter (Thermo-Orion Inc., Waltham, MA, USA).

### 2.3. Isolation of Endophytic Fungi

Endophytic fungi were isolated from fresh plant roots, which were disinfected by soaking in 75% ethanol (0.5 min), 3% sodium hypochlorite (5–8 min) and 75% ethanol (1 min) and were then rinsed twice for 1 min in sterile water. The plant roots were cut into small pieces (0.5 cm) using a sterilized scalpel. Segments from each plant tissue were randomly chosen and placed in Petri dishes containing potato dextrose agar (PDA) supplemented with 50 µg·mL^−1^ streptomycin and 50 µg·mL^−1^ chloramphenicol. These plates were incubated at 28 °C until fungal growth appeared. The colonies were counted and grouped by their morphologic characteristics, and representative isolates of fungal diversity were collected, purified, and preserved for future analysis.

### 2.4. Identification of Isolated Fungi

The isolated fungi were grown for 7 to 10 days on plates of potato-dextrose agar (PDA) in an incubator at 25 °C. After incubation, cultures were examined for color, margin, and texture of colonies in the agar medium, and were preliminarily identified based on morphological characteristics. For DNA extraction, mycelia were transferred from PDA into 250 mL Erlenmeyer’s flasks containing potato-dextrose broth without shaking. After 5 days of growth at 28 ± 2 °C, approximately 100 mg of the mycelial biomass was harvested. The genomic DNA was isolated using the Qiagen DNeasy Mini Kit according to the manufacturer’s instructions. The internal transcribed spacer (ITS) regions of rDNA were amplified with universal primers ITS4 (5′-TCCTCCGCTTATTGATATGC-3′) and ITS5 (5′-GGAAGTAAAAGTCGTAACAAGG-3′). The PCR products were purified and sequenced by Sangon Biotech (Shanghai) Co., Ltd., China. The resulting ITS sequences were searched for homology with other genera and species deposited in the GenBank database using NCBI BLAST analysis (http://www.ncbi.nlm.nih.gov/BLAST/ (accessed on 16 October 2021)). The phylogenetic reconstruction was calculated using the neighbor-joining (NJ) algorithm and the maximum-parsimony (MP) method. NJ analysis was conducted using the MEGA version 6.0 software with bootstrap values calculated from 1000 replicate runs. MP analysis was performed using the PAUP version 4.0 beta 10 program with bootstrap values (1000 replicate runs).

### 2.5. High-Throughput Sequencing and Data Processing

The genomic DNA of the soil samples (in summer and winter) was extracted using the Mag-Bind Soil DNA Kit Protocol (OMEGA, Norcross, GA, USA). The rDNA region was amplified by polymerase chain reaction (PCR) using the ITS1F (5′-CTTGGTCATTTAGAGGAAGTAA-3′) and ITS4 (5′-TCCTCCGCTTATTGATATGC-3′). Single-molecule real-time (SMRT) sequencing on PacBio Sequel II platforms was employed to generate circular consensus sequences (CCS) according to the standard protocols by Biomarker Technologies Corporation Co., Ltd. (Beijing, China). CCS reads are filtered, clustered, and de-noised to generate full-length amplicon tags for species annotation and abundance analysis.

Further analysis including diversity analysis, differential analysis between groups, correlation analysis, function prediction, etc. were carried out using the tools on the platform BMKCloud (www.biocloud.net (accessed on 12 October 2021)). Fungi Functional Guild (FUNGuild) was used to analyze fungal OTU by taxonomy of ecological guilds, and classify large sequence libraries into categories with ecological significance. Non-Metric Multi-Dimensional Scaling Ordination (NMDS) was used to visualize the potential differences (based on the Jaccard matrix) in the soil fungal community structure between groups or within groups. Significance of differences between sample clusters was assessed with Analysis of Similarity (ANOSIM). Random Forest (RF) models were generated, which provides the initial ranking of the genera according to its importance by the mean decrease Gini. Then, the top 30 genera were selected and their abundance was presented in the heatmap. Linear discriminant analysis Effect Size (LEfSe) analysis was conducted to identify and compare unique fungal taxa significantly related to each site at the different seasons. The biomarkers fungal groups were depicted in cladograms, and linear discriminant analysis (LDA) scores ≥4.0 were then performed. Spearman rank correlation analysis was processed based on the abundance of each species in the investigated sites. The correlation network was constructed with the ones with correlation larger than 0.1 and *p*-value smaller than 0.05. Canonical Correspondence analysis (CCA) was used to reflect the relationship between the fungi (top 10 genera and the key genera identified by random forest, LefSe, and co-occurrence analysis) at the genus level and environmental variables. Environmental variables were chosen only when their addition did not cause an inflation factor of variance >20.

### 2.6. Statistical Analysis

Two-way analysis of variance (ANOVA) was used to determine the individual and combined effects of sites distribution and sampling seasons on the soil physicochemical properties using IBM SPSS 25.0 (IBM Inc., Chicago, IL, USA).

## 3. Results and Discussion

### 3.1. Soil Physicochemical Properties

A two-way ANOVA indicated that the physicochemical parameters (i.e., the EC values and contents of SN and SC) of the soil samples differed significantly (*p* < 0.05) among the three sampling sites (i.e., sites F, H, and T) and between the two seasons (summer and winter 2020) (Table 1). There was a significant difference in pH values of the soil samples between two seasons (*p* < 0.05). A significant interaction term (i.e., sampling sites × seasons) for EC value and SC content (*p* < 0.05) was also detected. In both seasons, the average percentages of TN and SC content in the rhizosphere soil samples were gradually decreased in the order of sites F, H, and T. The salinity level (EC value) in the rhizosphere soils was the highest at site H, while it was not so high as expected at site T.

The investigated sites were located in the coastal area of Nantong, Jiangsu Province of China. There is a long history of the coastal mudflats’ reclamation in this area. Although the detailed reclamation ages of the sampling sites were unknown, the vertical distance from the location of the sites (in the order of sites F, H, and T) to the coastline could indicate a chronosequence reclamation of coastal mudflat. If the coastal areas were reclaimed at different stages, the impact of reclamation on soil properties could be varied with each other [2,27]. In general, the average percentages of soil SN and SC contents increased with increasing reclamation ages. At site T, the low EC value could be due to the desalination measures employed in the newly reclaimed land.

### 3.2. Soil Fungal Diversity and Community Structure

In the soil samples, the species diversity was evaluated using the Shannon and Simpson index, while ACE and Chao1 indexes were used to reflect the species richness of fungi communities (Table 2). In terms of the fungal diversity and richness in the investigated area, the diversity of fungi was highest at site F, followed by the sites H and T, and the index values were higher in winter compared with those in summer.

**Figure 1:**
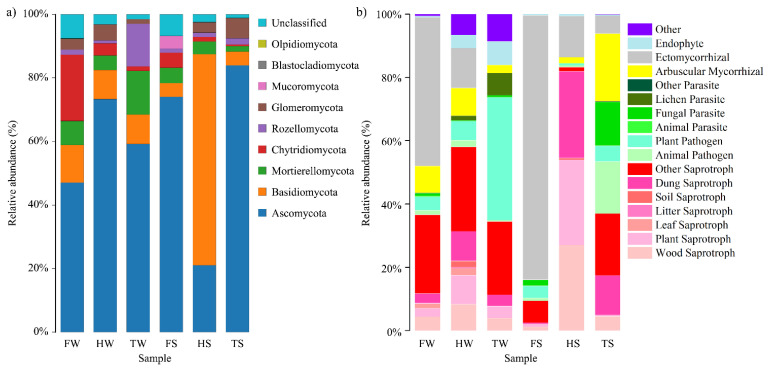
Bacterial community composition at the phylum level (**a**) and the relative abundances of fungal functionally guilds (**b**).

In this study, six major phyla were identified in all collected soil samples, including the phyla *Ascomycota*, *Basidiomycota*, *Mortierellomycota*, *Chytridiomycota*, *Rozellomycota*, and *Glomeromycota* (Figure 1a). Except for the sample HS, the majority of OTUs in the investigated sites were belonging to phylum *Ascomycota*. In the sample HS, the highest abundance of phylum *Basidiomycota* was found, and its abundance was even higher than phylum *Ascomycota*. The findings that *Ascomycota* and *Basidiomycota* were predominant phyla in the sampling sites were consistent with an investigation on the reclaimed land in the Sanjiang plain, China [11].

FUNGuild was used to examine the fungal community from an ecological perspective, which could explore fungal trophic type and metabolic function characteristics rather than taxonomic identity [28]. Based on FunGuild ecological guild assignments (Figure 1b), the highest relative abundances of AM fungi and endophytic fungi were observed in samples TS (21.4%) and TW (7.46%), respectively. A large proportion of the OTUs at site F belonged to ECM fungi, and the relative abundances of ECM fungi were 47.0% and 83.5% for samples FW and FS, respectively. According to the location of the sampling sites, AM fungi and endophytic fungi were more frequent at the sites close to the sea embankment (i.e., TS and TW, respectively), while ECM fungi were dominant at the site located in the protection forest along the highway (i.e., FS). The difference in relative abundances of AM and ECM fungi among the investigated sites was probably due to the different vegetation types. In general, AM fungi were prevalent in herbaceous plants, while ECM prefer to form symbiosis with temperate woody plant species [29]. Additionally, AM fungi generally establish symbiotic associations with plants in the early stages of growth, while ECM fungi dominate in mature plants [9,21,30]. The establishment of a dense woody plant (i.e., 5-year-old willow) cover at site F could explain the dominance of ECM. At the site close to a farm along the coastal area (i.e., samples HS and HW), FUNGuild database analyses revealed a higher proportion of saprophytic fungi. The relative abundance of dung saprotroph, plant saprotroph, and wood saprotroph were 27.4%, 26.9%, and 27.1%, respectively, in the HS sample, indicating a disturbance from agricultural industry. It is noteworthy that plant pathogens accounted for 38.8% abundance of the fungal OTUs in sample TW, which cannot be ignored due to potential harmful effects on plant health.

### 3.3. Isolation of Endophytic Fungi from Plant Roots

A total of 29 endophytic fungal taxa were isolated from the roots of the plant distributed in the investigated sites. Among them, genera *Alternaria*, *Fusarium*, *Monosporascus*, and *Podospora* were the four most common genera that were detected in the roots of many plant species (Table S1). *Alternaria* was observed mostly in willow, while *Fusarium*, *Monosporascus*, and *Podospora* were mostly isolated in plant roots from site H. Besides the four genera, *Aspergillus*, *Aureobasidium*, *Cladosporium*, *Clonostachys*, *Dactylonectria*, *Diaporthe*, *Mortierella*, *Penicillium*, *Sarocladium*, *Stemphylium*, and *Talaromyces* were detected by both root isolation and soil ITS sequencing. According to Hardoim et al. [31], the endophytic community can be classified into facultative endophytes, obligate endophytes, and passenger endophytes. The aforementioned genera belonged to the facultative endophytes that can live inside the host plant and the soil habitats. Strains from genera *Aspergillus*, *Penicillium*, and *Talaromyces* were distributed in the plant roots with low frequency of appearance. However, they were widely distributed with relatively low abundance in soil samples according to the ITS sequencing results. These three genera might belong to passenger endophytes, which randomly enter the plant. The fungi that were isolated from the plant roots but were not detected in the soil samples via ITS sequencing (*Arthrobotrys*, *Botrytis*, *Macrophomina*, *Paecilomyces*, *Parengyodontium*, *Peniophora*, *Phaeomyces*, *Phialophora*, *Phoma*, *Pithomyces*, and *Rhizoctonia*) could be classified into obligate endophytes.

Among the isolated fungi, genera *Alternaria* [32], *Aspergillus* [33], *Cladosporium* [34], *Phoma* [35], and *Phialophora* [36] contain strains classified as dark septate endophytes (DSE). Positive effects of DSE on the growth of host plants were reported, including the production of bioactive metabolites against pathogens [37], the synthesis of phytohormones, and the mineralization of organic N-containing compounds [38]. Additionally, strains from the genera *Aspergillus*, *Aureobasidium*, *Cladosporium*, *Monosporascus*, and *Sarocladium* have been reported as halophilic fungi [39]. Among them, genera *Aspergillus*, *Aureobasidium*, and *Monosporascus* were only isolated in plant roots from site H. Strains from the genera *Mortierella*, *Penicillium*, and *Talaromyces* have been recognized as plant growth promoting fungi [40,41]. The presence of the aforementioned fungal endophytes in the plant roots from the investigated sites indicates their important role in plant–fungus interactions. Nevertheless, previous literature showed that the majority of the genera listed (Table S1) include pathogenic fungal strains [42,43]. Among them, genus *Fusarium* is widely accepted as one of the major groups of soil-borne root pathogenic fungi [44]. In vitro tests need to be employed to evaluate the pathogenicity and confirm the function of the isolated fungi.

### 3.4. Spatial and Temporal Variation of Soil Fungal Communities

**Figure 2:**
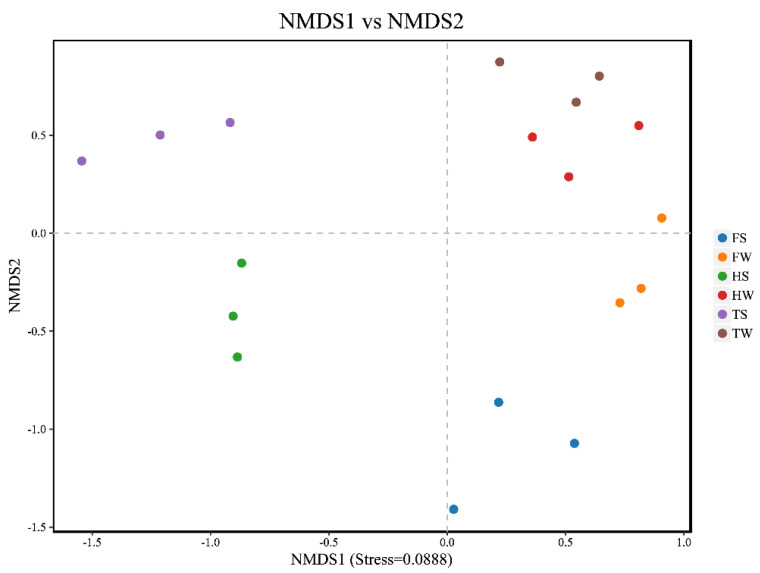
Non-metric multidimensional scaling plot for soil fungal communities at the genus level.

As the EC values and the average percentages of SN and SC contents in the soil samples differed significantly among the sampling sites and between the seasons, it was expected that these factors would be the key drivers for the distribution of fungi community. Indeed, the NMDS plot showed that the samples collected at different sites in two seasons were separated from each other, and the ANOSIM revealed a significant difference among them (*R* = 0.983, *p* = 0.001) (Figure 2). Seasonality is commonly regarded as an important parameter influencing plant-associated microbial communities [45]. In this study, the samples collected at different seasons were separated from each other. Besides providing essential nutrition (e.g., carbon and nitrogen source) for microbial growth, soil also affects the growth and distribution of microbe by its physicochemical properties (e.g., EC and pH) [10]. The samples collected at site F (distributed at the bottom right of the coordinate plane) were separated from those sampled at sites H and T. At site F, a dense tree canopy and relatively lower EC value and SN and SC contents were observed. The preferable environmental conditions could promote the dominance of ECM fungi, which explain the clustering pattern of the samples in the NMDS plot.

**Figure 3:**
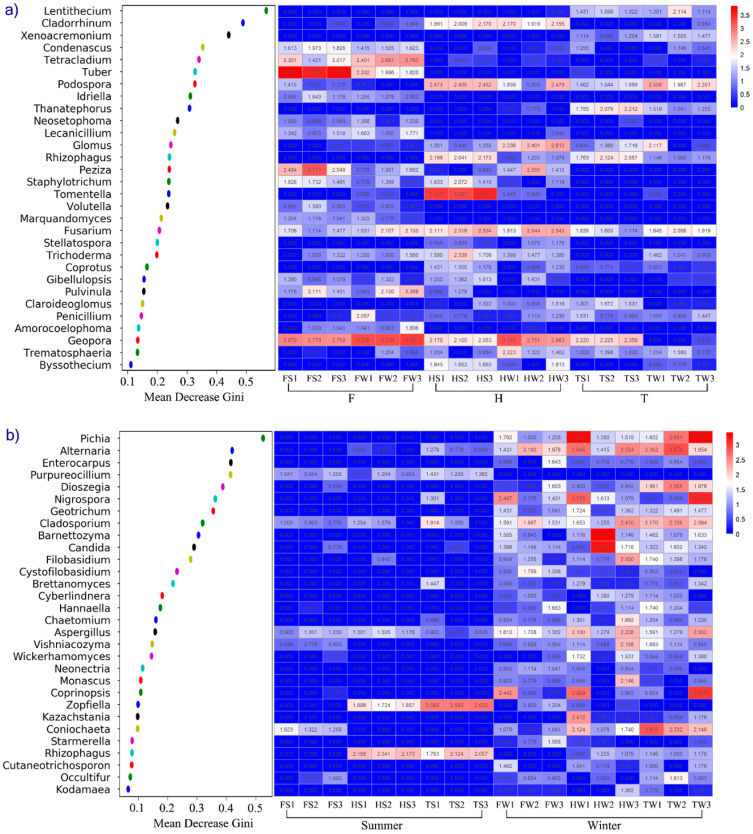
Top 30 genera with the highest Gini score according to the random forest analysis (investigated sites (**a**) and seasons (**b**)) were chosen to create the heat map. The abundances of these genera were converted to log10(x + 1) values.

The random forest analysis was performed to identify the signature microbiota in response to the variation of the investigated sites and seasons, respectively. It is expected that random-forest analysis could be used to classify the fungi community as mirroring sampling sites and seasons, suggesting essential factors shaping soil fungi community. Figure 3 shows the result of the random forest classification preformed using genera-level composition data of the rhizosphere soil samples. The abundance of fungi genera with the 30 highest Gini scores in the random forest analysis is presented in the heatmap. Among these selected genera, four dominant genera (*Tuber*, *Peziza*, *Tomentella*, and *Geopora*) belonging to ECM fungi were the potential contributors to identify the sites at which rhizosphere soil samples were collected (Figure 3a). ECM fungi were abundant in the FS (*Tuber*) and HS (*Tomentella*) samples, while it was seldom observed at site T. The site T located in a young reclaimed region, in which the C% and N% contents of the soil samples were the lowest. Five genera (*Pichia*, *Barnettozyma*, *Candida*, *Nigrospora*, and *Coprinopsis*) with high abundance in winter contributed to distinguishing the winter samples from the summer samples (Figure 3b), among which three genera (*Pichia*, *Barnettozyma*, and *Candida*) were identified as yeast, and their dominance was observed in winter.

**Figure 4:**
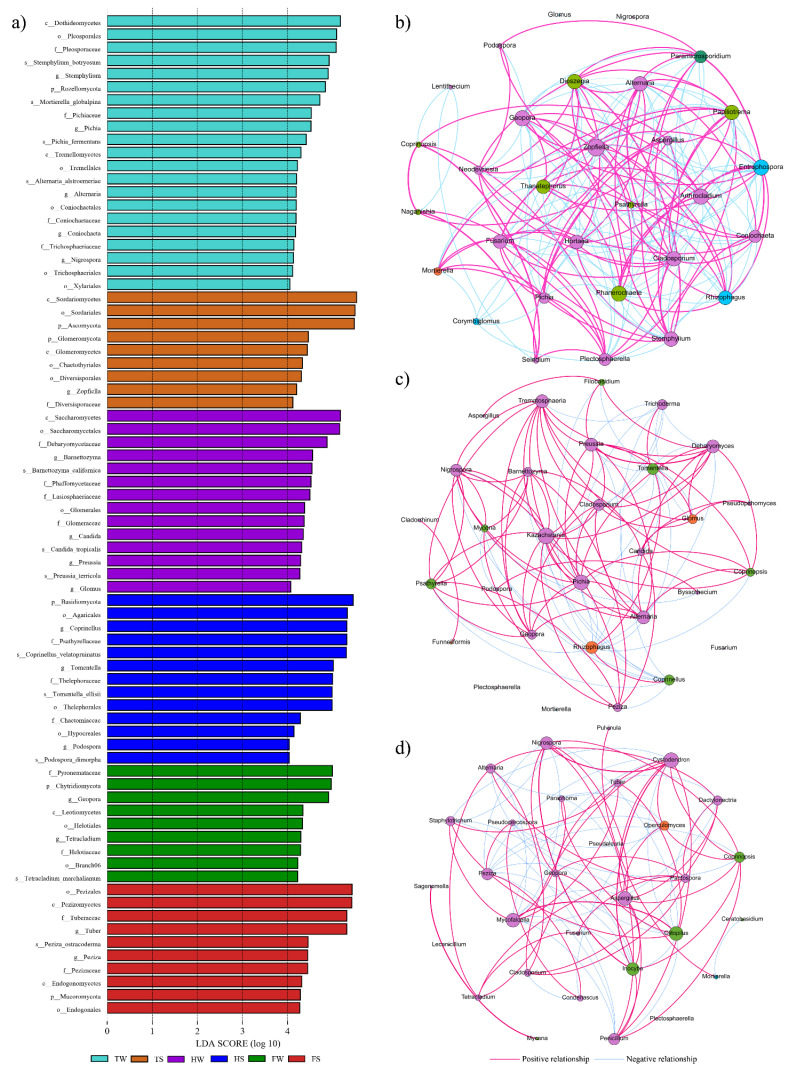
(**a**) LDA scores of the identified biomarkers in the six samples by linear discriminant analysis effect size pipeline analysis. (**b**–**d**) Co-occurrence network interactions of the bacterial communities in the investigated sites (i.e., T (**b**), H (**c**), and F (**d**)). Connections represent strong (Spearman’s *p* > 0.8) and significant (*p* < 0.01) correlations. Node size is proportional to the number of connections.

LEfSe analysis (LDA threshold 4.0) was conducted to identify and compare unique fungal taxa in the soil samples with statistical difference among different groups (Figure 4a). For samples from site F, fungi that were differentially abundant include genera *Tuber* and *Peziza* in the FS sample and genera *Geopora* and *Tetracladium* in the FW sample. At site H, biomarkers mainly comprised of genera *Podospora*, *Coprinellus* and *Tomentella* in the sample HS and genera *Preussia*, *Glomus* and *Candida* in the sample HW. At site T, genus *Zopfiella* was the biomarker in the TS sample, while the biomarkers in the TW sample include genera *Nigrospora*, *Coniochaeta*, *Alternaria*, *Stemphylium*, and *Pichia*.

A network interface was constructed to show the topological and taxonomic characteristics of the fungal co-occurrence patterns in each region (Figure 4b–d). The densities of the co-occurrence network at sites F, H, and T were 0.189, 0.241, and 0.409, respectively. These results suggested that the fungal network at site T was more connected than that at sites F and H. Nodes with high degree values and centrality metrics (betweenness or closeness centrality) were recognized as keystone species in the co-occurrence network. The keystone species at sites F, H, and T were identified, which could play critical roles in the co-occurrence network. Genera *Clitopilus* and *Aspergillus* were identified as the keystone genera at site F. At site H, keystone genera including *Rhizophagus*, *Nigrospora*, *Pichia*, and *Kazachstania* were identified. Genera *Papiliotrema*, *Phanerochaete*, *Entrophospora*, and *Zopfiella* were recognized as the keystone taxa at site T.

In the FS sample, the relative abundances of genera *Tuber* and *Peziza* were the highest. Genus *Geopora* was detected in most of the investigated sites, with the highest abundance in the sample FW. Genera *Tuber*, *Peziza*, and *Geopora* belong to order *Pezizales*. Many species in order *Pezizales* were recognized as ECM fungi that grow in symbiosis with the plant roots [46]. Both RF and LEfSe analyses indicated that genus *Tuber* had a preference for its distribution among the three sites. At site F, the higher soil SC and SN contents and lower EC values compared with the other two sites could be responsible for the dominance of genus *Tuber*. As the keystone genera at site F, genus *Clitopilus* was only detected in sample FS, while genus *Aspergillus* was identified in all samples. Genus *Clitopilus* belongs to family *Entolomataceae*, in which many strains can form ectomycorrhizae with a wide range of tree species; being considered as a common saprotrophic genus, it was reported to increase plant growth via facilitated potassium uptake [47]. Genus *Aspergillus* was widely distributed in all the investigated sites. Liang et al. [48] reported a ribosomal protein from halophilic strain (*Aspergillus glaucus*) that could confer salt tolerance in heterologous organisms. It is noteworthy that genus *Aspergillus* was recognized as a culturable fungal endophyte, and it was only isolated from the plant roots at site H. Genus *Tetracladium* was mainly identified at site F, with a higher abundance in the sample FW than that in the sample FS. Sati and Pant [49] reported a *Tetracladium* strain isolated from healthy roots of *Berberris vulgaris* growing in a riparian area had the potential to solubilize phosphate. As an endophytic fungus, genus *Tetracladium* could be capable of promoting plant growth at site F.

In the sample HS, the highest abundance of genus *Podospora* was detected. Genus *Coprinellus* were dominant in the sample HS, while they were seldom observed in other samples. The abundance of genus *Preussia* in winter was higher than that in summer, and its highest abundance were observed in the sample HW. Genera *Podospora*, *Coprinellus*, and *Preussia* include coprophilous species inhabiting the dung of various herbivores [50,51,52]. The site H is close to a poultry farm, which might probably release poultry excrement to the surrounding environment. It could explain why the coprophilous species were dominant at site H. In the sample HS, genus *Tomentella* were dominant. It was commonly found as an ectomycorrhizal partner of many trees, such as willow, birch, or alder, with the tendency to dominate in unfavorable environmental conditions (e.g., salinity stress) [46]. The dominance of genera *Glomus* and *Rhizophagus* (belonging to AM fungi) was observed in HW and HS, respectively. *Glomus* species was reported to increase the number of fruits and yield in papaya plants [53], while genus *Rhizophagus* could stabilize soil aggregates, enhance plant nutrition, and improve plant growth [16]. AM fungi form a mutualistic association with the roots of 90% of the terrestrial plants, and the symbiosis between AM fungi and plant roots is a well-recognized beneficial interaction occurring in soil [54]. They were reported to be capable of improving rooting, enhancing plant nutrition, favoring nutrient renovation, and promoting tolerance to biotic and abiotic stress [53]. Unfortunately, the use of AM fungi as an inoculant on a large scale is not yet widely used because of several difficulties in obtaining a large amount of inoculum (e.g., low growth and high competition with native AM fungi) [16]. Genera *Candida*, *Pichia*, and *Kazachstania* were described as ascomycetous yeast species [55,56]. Yeasts are particularly suitable as biocontrol agents against several kinds of rot fungi because their activity does not usually rely on the production of toxic metabolites, but rather on their ability to compete with pathogens for space and nutrients [57]. Some species belonging to genus *Candida* were reported to stimulate rice seedling growth [58]. Their dominance at site H (especially in winter) could be beneficial for the plants to defend against pathogens and survive under the salinity stress. However, a *Pichia* strain (*Pichia fermentans*) with dual activity (an effective biocontrol agent and an aggressive pathogen) was reported [55]. To avoid unpredictable effects, it needs a thorough risk analysis before the application of yeast species as biocontrol agents. Many strains in the genus *Nigrospora* are characterized as plant endophytic micro-organisms. This genus produces a broad range of bioactive secondary metabolites, which could be used to inhibit the growth of plant pathogenic fungi [59].

In the sample TS, the dominance of genus *Entrophospora* was observed. It was classified as an AM fungus [53], which could exchange nutrients gathered from soil for carbon compounds provided by plants. They are important microbial symbionts for plants, especially when soil total phosphorus (TP) and TN are limiting [60]. Although soil TP content was not measured in this study, the lower SC and SN contents at site T indicated a possible deficit in TP contents. The appearance of genus *Entrophospora* might favor plant growth at site T. Genera *Nigrospora* and *Coniochaeta* were observed in winter, especially in the sample TW. Genus *Zopfiella* was observed with the highest abundance in the sample TS. Some strains of the genera *Nigrospora*, *Coniochaeta*, and *Zopfiella* are characterized as plant endophytic micro-organisms, and they are capable of promoting the stress resistance of the host plant and producing antifungal compounds [61,62,63]. Genera *Alternaria* and *Stemphylium* include both plant-pathogenic and saprophytic species [64,65]. Their dominance in winter, especially in the sample TW, indicated possible damage or decay of plants after establishing pathogenic, saprotrophic, or endophytic relationships with the plant hosts. Additionally, genus *Phanerochaete* that was recognized as a saprotrophic fungus appeared in the sample TS. It is capable of degrading lignin and mineralizing a wide variety of priority aromatic pollutants [66].

### 3.5. The Relationship between Fungi Genera and Soil Physical-Chemical Parameters

**Figure 5:**
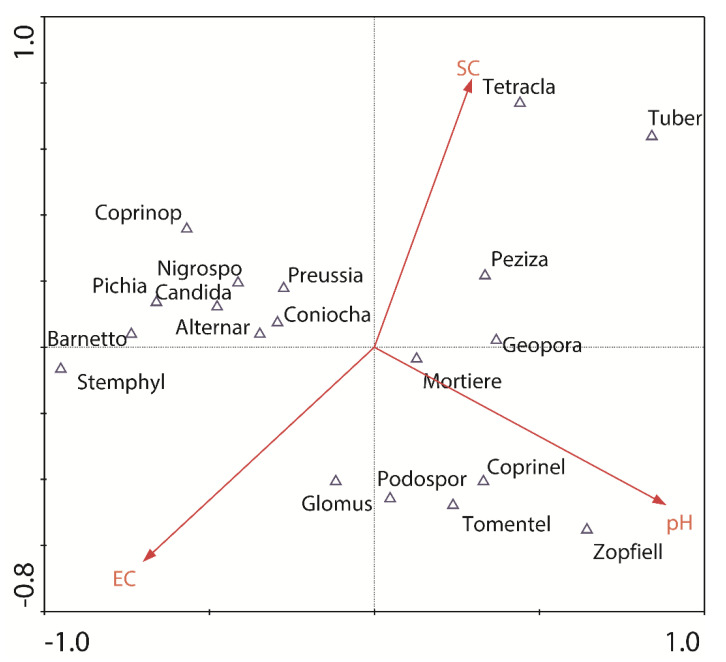
Two−dimensional ordination diagram of fungi-environmental factors. Canonical correspondence analysis was used to reflect the relationship between the fungi (top 10 dominant genera and the genera identified by random forest, LefSe, and co-occurrence analysis) and environmental factors.

The relationships between the fungi (top 10 dominant genera, biomarkers, and keystone taxa) and selected environmental factors were explored by CCA (Figure 5). The two CCA axes explained a substantial proportion of the variation (88.8%) in the fungi–environment relationship.

The abundance of *Tuber* and *Geopora* (belonging to ECM fungi) and *Peziza* (containing both ECM and saprotrophic fungi) was positively related with SC content and negatively related with EC level. High soil salinity was generally recognized to have a negative impact on ECM associations [46]. Genera *Tuber* and *Geopora* were dominant at site F, which was characterized by high SC and SN contents and low soil EC value. ECM fungi are capable of excreting oxidative and hydrolytic enzymes to break down soil organic matter and liberate unavailable nutrients [8,67]. Their predominance (e.g., *Tuber* and *Geopora*) at site F could be helpful to transfer nutrients from organic matter to their host tree. There are also some ECM fungi that are highly adapted to saline conditions [10], and their appearance (e.g., *Tomentella*) at site H (with relatively high values of soil EC) was observed. Additionally, the positive relationship between the abundance of *Tetracladium* and SC content was also observed. Some strain (*Tetracladium setigerum*) belonging to *Tetracladium* was reported as a phosphate solubilization fungi, which has the potential to solubilize various source of phosphates through the production of phytases and phosphatases enzymes and the organic acids [49]. The dominance of genus *Tetracladium* at site F could provide sufficient P for the good development of shelter forest.

Soil pH value has a positive influence on the distribution of the genera *Zopfiella*, *Tomentella*, and *Coprinellus* that were dominant in summer during the investigation. Zhao et al. [68] reported that genera *Zopfiella* was dominated in the soil with the highest pH values after disinfestation treatment using bean dregs. During the investigation on fungi in protected coastal *Salix* repens communities in the Netherlands, Geml et al. [60] reported that *Tomentella* showed a strong preference for alkaline soils. Liu et al. [69] indicated that *Coprinellus* is preferably dwelling alkaline environments. Additionally, several yeasts (*Candida*, *Pichia*, and *Barnettozyma*) were negatively related with pH values. Birkhofer et al. [70] investigated the soil yeast community composition and abundance in different land use types in Germany, and reported that yeasts were highly abundant in the region with a low soil pH and high soil moisture.

There was a positive relationship between the EC value and the abundance of genera *Stemphylium* and *Glomus*. Gonçalves et al. [13] isolated *Stemphylium* from the field-collected *Salicornia*, and found that the growth of *Stemphylium* sp. was unaffected by the presence of NaCl (200–800 mM) in the growth medium. Furthermore, the inoculation of *Salicornia* with the isolated *Stemphylium* strain positively influenced total biomass production and N concentration in roots in salinity conditions (150 mM NaCl). It is already known that AM fungi (e.g., *Glomus*) widely exist in salt-affected soils, and they were reported to play a vital role in mitigating the adverse effects of salinity on plants by the processes including the up-regulation of the antioxidant system, the modulation of the biosynthesis of osmoprotectants, and the compartmentalization of excessive toxic ions into the vacuole [71,72,73].

## 4. Conclusions

The differences in soil physicochemical parameters (i.e., the EC values and contents of SN and SC), fungal diversity, and community structure were detected among the sampling sites (i.e., sites F, H and T) and between the seasons (summer and winter 2020). Based on FunGuild ecological guild assignments, the highest relative abundances of Ectomycorrhizal (ECM) fungi (83.5%), arbuscular mycorrhizal (AM) fungi (21.4%), and endophytic fungi (7.46%) were observed in the samples FS, TS, and TW, respectively. Genera *Tuber* and *Geopora* that belong to ECM fungi were dominant at site F, which was characterized by high SC and SN contents and low soil EC value. AM fungi, including genera *Glomus*, *Rhizophagus*, and *Entrophospora*, were dominant in the samples HW, HS, and TS, respectively. The positive relationship between the EC value and the abundance of genus *Glomus* indicated the ability of AM fungi to protect plants against the salt stress. According to LEfSe analysis and co-occurrence analysis, some biomarkers or keystone taxa belonging to endophytic fungi were recognized at sites F (*Aspergillus* and *Tetracladium*), H (*Nigrospora*), and T (*Nigrospora*, *Coniochaeta* and *Zopfiella*), which could contribute to the promotion of plant growth. However, most of them were not isolated from the plant roots in the investigated sites. A total of 15 endophytic fungi were detected by both root isolation and ITS sequencing of soil samples. It deserves further investigation to isolate more endophytic fungal strains from the plant roots and to evaluate their potential function in conferring stress tolerance to plants.

## Figures and Tables

**Table 1:** Description of the study sites.

Sampling Sites	F		H		T	
Latitude/Longitude	32.603° N, 120.880° E		32.609° N, 120.933° E		32.155° N, 121.475° E	
Vegetation Information	a dense tree canopy		a sparse tree layer and dense herb and shrub layers		sparse tree, herb and shrub layers	
Soil properties						
Seasons	Summer	Winter	Summer	Winter	Summer	Winter
pH	9.58 ± 0.05	8.84 ± 0.24	9.53 ± 0.27	8.92 ± 0.03	9.79 ± 0.17	8.70 ± 0.17
EC (μS/cm)	104.3 ± 33.2	129.3 ± 26.1	619.7 ± 217.9	2668.0 ± 321.2	384.8 ± 45.4	1005.0 ± 136.4
SN (%)	0.07 ± 0.02	0.12 ± 0.02	0.05 ± 0.01	0.06 ± 0.02	0.04 ± 0.01	0.05 ± 0.01
SC (%)	1.44 ± 0.19	1.80 ± 0.12	1.25 ± 0.06	1.32 ± 0.09	1.23 ± 0.03	1.17 ± 0.05

**Table 2:** The species diversity and richness index of the fungal community.

Sampling Sites	Seasons	Diversity Index		Richness Index	
		Shannon	Simpson	ACE	Chao1
F	Summer	3.901 ± 0.312	0.750 ± 0.061	192.310 ± 22.377	194.241 ± 22.156
	Winter	5.599 ± 0.129	0.947 ± 0.010	306.042 ± 21.626	309.471 ± 23.004
H	Summer	3.446 ± 0.030	0.747 ± 0.009	142.355 ± 5.127	144.526 ± 4.278
	Winter	4.959 ± 0.598	0.913 ± 0.037	269.587 ± 11.640	271.892 ± 8.320
T	Summer	2.653 ± 0.135	0.535 ± 0.028	121.253 ± 11.840	121.558 ± 12.478
	Winter	4.778 ± 0.401	0.906 ± 0.021	238.112 ± 33.987	240.534 ± 34.843

## Data Availability

We have deposited the sequencing data in NCBI with accession number PRJNA800618. Other data presented in this study are available in Supplementary Materials.

## Supplementary Materials

The following supporting information can be downloaded at: https://www.mdpi.com/article/10.3390/jof8020124/s1, Table S1: The fungal strains isolated from the plant roots in the investigated sites.

## References

[1] Zhang X., Liao X., Huang L., Shan Q., Hu A., Yan D., Zhang J., Long X.E. (2021). Soil profile rather than reclamation time drives the mudflat soil microbial community in the wheat-maize rotation system of Nantong, China. J. Soils Sediments.

[2] Li J., Pu L., Zhu M., Zhang J., Li P., Dai X., Xu Y., Liu L. (2014). Evolution of soil properties following reclamation in coastal areas: A review. Geoderma.

[3] Chung C.H., Zhuo R.Z., Xu G.W. (2004). Creation of *Spartina plantations* for reclaiming Dongtai, China, tidal flats and offshore sands. Ecol. Eng..

[4] Cui X., Hu J., Wang J., Yang J., Lin X. (2016). Reclamation negatively influences arbuscular mycorrhizal fungal community structure and diversity in coastal saline-alkaline land in Eastern China as revealed by Illumina sequencing. Appl. Soil Ecol..

[5] Rozema J., Flowers T. (2008). Ecology crops for a salinized world. Science.

[6] Colmer T.D., Pedersen O., Wetson A.M., Flowers T.J. (2013). Oxygen dynamics in a salt-marsh soil and in *Suaeda maritima* during tidal submergence. Environ. Exp. Bot..

[7] Li J.L., Sun X., Zheng Y., Lu P.P., Wang Y.L., Guo L.D. (2020). Diversity and community of culturable endophytic fungi from stems and roots of desert halophytes in northwest China. MycoKeys.

[8] Teste F.P., Jones M.D., Dickie I.A. (2020). Dual-mycorrhizal plants: Their ecology and relevance. New Phytol..

[9] Wilgan R., Shrivastava N., Mahajan S., Varma A. (2021). Dual and Tripartite Symbiosis of Invasive Woody Plants. Symbiotic Soil Microorganisms.

[10] Thiem D., Golebiewski M., Hulisz P., Piernik A., Hrynkiewicz K. (2018). How does salinity shape bacterial and fungal microbiomes of *Alnus glutinosa* roots?. Front. Microbiol..

[11] Xu F., Cai T., Yang X., Sui W. (2017). Soil fungal community variation by large-scale reclamation in Sanjiang plain, China. Ann. Microbiol..

[12] Fernandes E.G., Pereira O.L., da Silva C.C., Bento C.B., de Queiroz M.V. (2015). Diversity of endophytic fungi in *Glycine max*. Microbiol. Res..

[13] Gonçalves D.R., Pena R., Zotz G., Albach D.C. (2021). Effects of fungal inoculation on the growth of *Salicornia* (*Amaranthaceae*) under different salinity conditions. Symbiosis.

[14] Wang F.Y., Liu R.J., Lin X.G., Zhou J.M. (2004). Arbuscular mycorrhizal status of wild plants in saline-alkaline soils of the Yellow River Delta. Mycorrhiza.

[15] Chen J., Zhang H., Zhang X., Tang M. (2017). Arbuscular mycorrhizal symbiosis alleviates salt stress in black locust through improved photosynthesis, water status, and K^+^/Na^+^ homeostasis. Front. Plant. Sci..

[16] Cely M.V., de Oliveira A.G., de Freitas V.F., de Luca M.B., Barazetti A.R., Dos Santos I.M., Gionco B., Garcia G.V., Prete C.E., Andrade G. (2016). Inoculant of arbuscular mycorrhizal fungi (*Rhizophagus clarus*) increase yield of soybean and cotton under field conditions. Front. Microbiol..

[17] Verbruggen E., van der Heijden M.G., Rillig M.C., Kiers E.T. (2013). Mycorrhizal fungal establishment in agricultural soils: Factors determining inoculation success. New Phytol..

[18] Fontana D.C., de Paula S., Torres A.G., de Souza V.H.M., Pascholati S.F., Schmidt D., Neto D.D. (2021). Endophytic fungi: Biological control and induced resistance to phytopathogens and abiotic stresses. Pathogens.

[19] Hardoim P.R., van Overbeek L.S., Berg G., Pirttila A.M., Compant S., Campisano A., Doring M., Sessitsch A. (2015). The hidden world within plants: Ecological and evolutionary considerations for defining functioning of microbial endophytes. Microbiol. Mol. Biol. Rev..

[20] Botnen S., Kauserud H., Carlsen T., Blaalid R., Høiland K. (2015). Mycorrhizal fungal communities in coastal sand dunes and heaths investigated by pyrosequencing analyses. Mycorrhiza.

[21] Thiem D., Piernik A., Hrynkiewicz K. (2017). Ectomycorrhizal and endophytic fungi associated with *Alnus glutinosa* growing in a saline area of central Poland. Symbiosis.

[22] Johansen R.B., Johnston P., Mieczkowski P., Perry P.L.W., Robeson M.S., Burns B.R., Vilgalys R. (2016). A native and an invasive dune grass share similar, patchily distributed, root-associated fungal communities. Fungal Ecol..

[23] Simoes M.F., Antunes A., Ottoni C.A., Amini M.S., Alam I., Alzubaidy H., Mokhtar N.A., Archer J.A.C., Bajic V.B. (2015). Soil and rhizosphere associated fungi in gray mangroves (*Avicennia marina*) from the red sea—A metagenomic approach. Genom. Proteom. Bioinf..

[24] Wang Y., Qiu Q., Yang Z., Hu Z., Tam N.F.Y., Xin G. (2009). Arbuscular mycorrhizal fungi in two mangroves in South China. Plant Soil.

[25] Zhang G., Bai J., Tebbe C.C., Huang L., Jia J., Wang W., Wang X., Yu L., Zhao Q. (2021). *Spartina alterniflora* invasions reduce soil fungal diversity and simplify co-occurrence networks in a salt marsh ecosystem. Sci. Total Environ..

[26] Schneider-Maunoury L., Deveau A., Moreno M., Todesco F., Belmondo S., Murat C., Courty P.E., Jkalski M., Selosse M.A. (2020). Two ectomycorrhizal truffles, *Tuber melanosporum* and *T. aestivum*, endophytically colonise roots of non-ectomycorrhizal plants in natural environments. New Phytol..

[27] Bai J., Xiao R., Zhang K., Gao H., Cui B., Liu X. (2013). Soil organic carbon as affected by land use in young and old reclaimed regions of a coastal estuary wetland, China. Soil Use Manag..

[28] Nguyen N.H., Song Z., Bates S.T., Branco S., Tedersoo L., Menke J., Schilling J.S., Kennedy P.G. (2016). FUNGuild: An open annotation tool for parsing fungal community datasets by ecological guild. Fungal Ecol..

[29] Veresoglou S.D., Wulf M., Rillig M.C. (2017). Facilitation between woody and herbaceous plants that associate with arbuscular mycorrhizal fungi in temperate European forests. Ecol. Evol..

[30] Puttsepp U., Roslingd A., Taylor A.F.S. (2004). Ectomycorrhizal fungal communities associated with *Salix viminalis* L. and *S. dasyclados* wimm. clones in a short-rotation forestry plantation. Forest Ecol. Manag..

[31] Hardoim P.R., van Overbeek L.S., Elsas J.D. (2008). Properties of bacterial endophytes and their proposed role in plant growth. Trends Microbiol..

[32] He C., Wang W., Hou J., Li X. (2021). Dark septate endophytes isolated from wild licorice roots grown in the desert regions of northwest China enhance the growth of host plants under water deficit stress. Front. Microbiol..

[33] Barrow J.R., Osuna P. (2002). Phosphorus solubilization and uptake by dark septate fungi in fourwing saltbush, *Atriplex canescens* (Pursh) Nutt. J. Arid Environ..

[34] Li X., He C., He X., Su F., Hou L., Ren Y., Hou Y. (2019). Dark septate endophytes improve the growth of host and non-host plants under drought stress through altered root development. Plant Soil.

[35] He C., Wang W., Hou J. (2020). Plant performance of enhancing licorice with dual inoculating dark septate endophytes and *Trichoderma viride* mediated via effects on root development. BMC Plant Biol..

[36] Newsham K.K. (1999). *Phialophora graminicola*, a dark septate fungus, is a beneficial associate of the grass *Vulpia ciliata* ssp. *Ambigua*. New Phytol..

[37] Chatterjee S., Ghosh R., Manda N.C. (2019). Production of bioactive compounds with bactericidal and antioxidant potential by endophytic fungus *Alternaria alternata* AE1 isolated from *Azadirachta indica* A. Juss. PLoS ONE.

[38] Newsham K.K. (2011). A meta-analysis of plant responses to dark septate root endophytes. New Phytol..

[39] Smolyanyuk E.V., Bilanenko E.N. (2011). Communities of halotolerant micromycetes from the areas of natural salinity. Microbiology.

[40] Yamagiwa Y., Inagaki Y., Ichinose Y., Toyoda K., Hyakumachi M., Shiraishi T. (2011). *Talaromyces wortmannii* FS2 emits β-caryphyllene, which promotes plant growth and induces resistance. J. Gen. Plant Pathol..

[41] Ozimek E., Hanaka A. (2021). *Mortierella* Species as the plant growth-promoting fungi present in the agricultural soils. Agriculture.

[42] Dean R., Kan J.A.L.V., Pretorius Z.A., Hammond-kosack K.E., Pietro A.D., Spanu P.D., Rudd J.J., Dickman M., Kahmann R., Ellis J. (2012). The top 10 fungal pathogens in molecular plant pathology. Mol. Plant Pathol..

[43] Crous P.W., Hawksworth D.L., Wingfield M.J. (2015). Identifying and naming plant-pathogenic fungi: Past, present, and future. Annu. Rev. Phytopathol..

[44] Michielse C.B., Rep M. (2009). Pathogen profile update: *Fusarium oxysporum*. Mol. Plant. Pathol..

[45] Shen S.Y., Fulthorpe R. (2015). Seasonal variation of bacterial endophytes in urban trees. Front. Microbiol..

[46] Hrynkiewicz K., Szymanska S., Piernik A., Thiem D. (2015). Ectomycorrhizal community structure of *Salix* and *Betula* spp. at a saline site in central Poland in relation to the seasons and soil parameters. Water Air Soil Pollut..

[47] Peng L., Shan X., Yang Y., Wang Y., Druzhinina I.S., Pan X., Jin W., He X., Wang X., Zhang X. (2021). Facultative symbiosis with a saprotrophic soil fungus promotes potassium uptake in American sweetgum trees. Plant Cell Environ..

[48] Liang X., Liu Y., Xie L., Liu X., Wei Y., Zhou X., Zhang S. (2015). A ribosomal protein AgRPS3aE from halophilic *Aspergillus glaucus* confers salt tolerance in heterologous organisms. Int. J. Mol. Sci..

[49] Sati S.C., Pant P. (2018). Evaluation of phosphate solubilization by root endophytic aquatic Hyphomycete *Tetracladium setigerum*. Symbiosis.

[50] Házi J., Nagy L.G., Vágvölgyi C., Papp T. (2010). *Coprinellus radicellus*, a new species with northern distribution. Mycol Prog..

[51] Massimo N.C., Devan M.M.N., Arendt K.R., Wilch M.H., Riddle J.M., Furr S.H., Steen C., U’Ren J.M., Sandberg D.C., Arnold A.E. (2015). Fungal endophytes in aboveground tissues of desert plants: Infrequent in culture, but highly diverse and distinctive symbionts. Microb. Ecol..

[52] Paoletti M., Saupe S.J. (2008). The genome sequence of *Podospora anserina*, a classic model fungus. Genome Biol..

[53] Vázquez-Hernández M.V., Arévalo-Galarza L., Jaen-Contreras D., Escamilla-García J.L., Mora-Aguilera A., Hernández-Castro E., Cibrián-Tovar J., Téliz-Ortiz D. (2011). Effect of *Glomus mosseae* and *Entrophospora colombiana* on plant growth, production, and fruit quality of ‘Maradol’ papaya (*Carica papaya* L.). Sci. Hortic..

[54] Smith S.E., Smith F.A. (2011). Roles of *arbuscular mycorrhizas* in plant nutrition and growth: New paradigms from cellular to ecosystem scales. Annu. Rev. Plant Biol..

[55] Fiori S., Scherm B., Liu J., Farrell R., Mannazzu I., Budroni M., Maserti B.E., Wisniewski M.E., Migheli Q. (2012). Identification of differentially expressed genes associated with changes in the morphology of *Pichia fermentans* on apple and peach fruit. FEMS Yeast Res..

[56] Limtong S., Yongmanitchai W., Tun M.M., Kawasaki H., Seki T. (2007). *Kazachstania siamensis* sp. nov., an ascomycetous yeast species from forest soil in Thailand. Int. J. Syst. Evol. Microbiol..

[57] Spadaro D., Vola R., Piano S., Gullino M.L. (2002). Mechanisms of action and efficacy of four isolates of the yeast *Metschnikowia pulcherrima* active against postharvest pathogens on apples. Postharvest Biol. Technol..

[58] Amprayn K.O., Rose M.T., Kecskés M., Pereg L., Nguyen H.T., Kennedy I.R. (2012). Plant growth promoting characteristics of soil yeast (*Candida tropicalis* HY) and its effectiveness for promoting rice growth. Appl. Soil Ecol..

[59] Saunders M., Kohn L.M. (2008). Host-synthesized secondary compounds influence the in vitro interactions between fungal endophytes of maize. Appl. Environ. Microbiol..

[60] Geml J., Gravendeel B., van der Gaag K.J., Neilen M., Lammers Y., Raes N., Semenova T.A., de Knijff P., Noordeloos M.E. (2014). The contribution of DNA metabarcoding to fungal conservation: Diversity assessment, habitat partitioning and mapping red-Listed fungi in protected coastal *Salix repens* communities in the Netherlands. PLoS ONE.

[61] Huang X., Liu L., Wen T., Zhu R., Zhang J., Cai Z. (2015). Illumina MiSeq investigations on the changes of microbial community in the *Fusarium oxysporum* f.sp. *cubense* infected soil during and after reductive soil disinfestation. Microbiol. Res..

[62] Kim J.C., Choi G.J., Park J.H., Kim H.T., Cho K.Y. (2001). Activity against plant pathogenic fungi of phomalactone isolated from *Nigrospora sphaerica*. Pest Manag. Sci..

[63] Kokaew J., Manoch L., Worapong J., Chamswarng C., Strobel G. (2011). *Coniochaeta ligniaria* an endophytic fungus from *Baeckea frutescens* and its antagonistic effects against plant pathogenic fungi. Thai J. Agric. Sci..

[64] Franco M.E.E., Lopez S.M.Y., Medina R., Lucentini C.G., Troncozo M.I., Pastorino G.N., Saparrat M.C.N., Balatti P.A. (2017). The mitochondrial genome of the plant-pathogenic fungus *Stemphylium lycopersici* uncovers a dynamic structure due to repetitive and mobile elements. PLoS ONE.

[65] Logrieco A., Moretti A., Solfrizzo M. (2009). *Alternaria toxins* and plant diseases: An overview of origin, occurrence and risks. World Mycotoxin J..

[66] Spadaro J.T., Gold M.H., Renganathan V. (1992). Degradation of azo dyes by the lignin-degrading fungus *Phanerochaete chrysosporium*. Appl. Environ. Microbiol..

[67] Nicolás C., Bertelsen T.M., Floudas D., Bentzer J., Smits M., Johansson T., Troein C., Persson P., Tunlid A. (2019). The soil organic matter decomposition mechanisms in ectomycorrhizal fungi are tuned for liberating soil organic nitrogen. ISME J..

[68] Zhao J., Zhou X., Jiang A., Fan J., Lan T., Zhang J., Cai Z. (2018). Distinct impacts of reductive soil disinfestation and chemical soil disinfestation on soil fungal communities and memberships. Appl. Microbiol. Biotechnol..

[69] Liu K., Ding X., Wang H.F., Zhang X., Hozzein W.N., Wadaan M.A., Lan A., Zhang B., Li W. (2014). Eukaryotic microbial communities in hypersaline soils and sediments from the alkaline hypersaline Huama Lake as revealed by 454 pyrosequencing. Antonie Leeuwenhoek.

[70] Birkhofer K., Schoning I., Alt F., Herold N., Klarner B., Maraun M., Marhan S., Oelmann Y., Wubet T., Yurkov A. (2012). General relationships between abiotic soil properties and soil biota across spatial scales and different land-use types. PLoS ONE.

[71] Tian C.Y., Feng G., Zhang F.S. (2004). Different effects of arbuscular mycorrhizal fungal isolates from saline or non-saline soil on salinity tolerance of plants. Appl. Soil Ecol..

[72] Hashem A., Abd_Allah E.F., Alqarawi A.A., Aldubise A., Egamberdieva D. (2015). Arbuscular mycorrhizal fungi enhances salinity tolerance of *Panicum turgidum* Forssk by altering photosynthetic and antioxidant pathways. J. Plant Interact..

[73] Porcel R., Aroca R., Ruiz-Lozano J.M. (2012). Salinity stress alleviation using arbuscular mycorrhizal fungi. A review. Agron. Sustain..

