# Supplementary material for: Soil Fungal Community Composition and Diversity of Culturable Endophytic Fungi from Plant Roots in the Reclaimed Area of the Eastern Coast of China
Source: J Fungi (Basel). 2022 Jan 27;8(2):124. doi: 10.3390/jof8020124 (PMC8878519; doi:10.3390/jof8020124)
Supplement: Supplementary file 1 [file jof-08-00124-s001.zip › jof-1541079-supplementary.pdf]

Table S1. The fungal strains isolated from the plant roots in the investigated sites.

	F		H										T	
	Salix matsudana		*Pyrus betulifolia*		*Suaeda glauca*		*Tamarix chinensis*		*Celtis sinensis*		*Salix matsudana*		*Salix matsudana*	*Tamarix chinensis*
	Summer	Winter	Summer	Winter	Summer	Winter	Summer	Winter	Summer	Winter	Summer	Winter	Summer	Winter
Alternaria	+										++	+		+
Arthrobotrys											+			
Aspergillus								+	+					
Aureobasidium						+								
Botrytis	+													
Cladosporium		+												+
Clonostachys														+
Dactylonectria	++													
Diaporthe							+							
Fusarium			+			+		+	++	+	+			
Macrophomina		+												
Monosporascus			+		+	+	++			+				
Mortierella									+					
Nectriaceae				+										
Paecilomyces								++						
Parengyodontium					+									
Penicillium														+
Peniophora	+													
Phaeomyces				+										
Phialophora										+				
Phoma			+											
Pithomyces												+		

Pleosporales				+									
Podospora			+			+	+				+		
Rhizoctonia													+
Sarocladium	+									+			
Sordariales				+									
Stemphylium	+												
Talaromyces												++	

+ (low) and ++ (high) indicate the frequency of the strains being isolated during the investigation.
